# Carbolic Acid in Scarlatina

**Published:** 1869-02

**Authors:** A. M. Carpenter

**Affiliations:** Keokuk, Iowa


### Carbolic Acid in Scarlatina.

BY A. M. CARPENTER, M. D., KEOKUK, IOWA.

I wish to add the weight of my testimony to the beneficial influences arising from the local use of the acid in the Anginose variety of scarlet fever. Not only does it materially lessen the inflammatory processes within the fauces, and conduce to the comfort of the little patient, but it prevents in some way, through its action upon the parts, septic poisoning, heals the ulcerated spots readily, and most effectually prevents the viscid faucial secretions from accumulating to an extent sufficient to embarrass the efforts at respiration. The tonsils, I have observed, resume their normal tint much sooner after its use than from any other local agent. Besides, the liability to consecutive abscess and otorrhœa has been diminished in a very marked degree. So, too, does the tendency to serous transudation seem to be obviated, due precaution being exercised against a change of temperature for three weeks succeeding the desquamative stage, at which time the liability to renal congestion is greatest. Within the past five weeks I have treated nineteen cases between the ages of one and ten years without a single fatal result. In eleven, the febrile movement reached its maximum intensity. Delirium and great restlessness, with stupor, obtained in all these cases—the range of the pulse 140 to 155 to the minute. The heat of the skin was lessened by constant sponging with cool or tepid water and vinegar, iced lemonade *ad libitum*, to allay thirst and soften the parched and swollen tongue, with occasional laxatives, and a solution of chlorate potassa, two drachms to the pint, as a drink, together with the following topical solution to the tonsils twice daily, (℞. Acid Carbol. f. ʒss, glycerine, Aq. distil. aa. ℥iss, misce) constituted the treatment.

To combat renal congestion spt. nit. dulc. in ten drop doses was relied on. Milk punch and animal essences were freely used when the heat of skin subsided. The acid assuredly supplies a void long felt by the profession; but let us hope that its value will neither be over estimated, nor the remedy discarded, until well tested.—*Physician and Pharmaceutist.*

